# Mosquitoes

**Published:** 1910-08-20

**Authors:** 


### Tropical Medicine.

### MOSQUITOES.

As a rule almost every summer one reads in a morning paper of the invasion of London by hordes of mosquitoes from the docks. It may astonish many to hear that this annual invasion, which does really appear if the weather is hot enough, is purely due to home-bred products; in short, is caused by indigenous English mosquitoes. Though it is true that tropical mosquitoes are from time to time brought home to this country in the holds of ships, they will not live in this climate unless specially cared for and heated, and they certainly never get the length of breeding. The mosquitoes that are found in London, and that bite quite readily, when the weather is hot, are ordinary English mosquitoes. It may still further astonish many when one states that numerous genera and species of real mosquitoes live in the British Isles, and not only in these climates, but even in the more northern regions of Siberia, Norway, and Russia. At the present moment the whole life-history of one of our ordinary English mosquitoes, *Culex pipiens*, is being passed in hundreds of gardens in London, the only essential requisite for this being, of course, water (water-butts or garden-tanks). In the swampy ground round East and West Ham another species abounds, for part of its life-history is passed in this special class of water, while in Kent, Buckinghamshire, parts of Surrey, Hampshire, and so on, real anopheles mosquitoes, exactly the same as those that spread malaria in Spain, Italy, Greece, and other places; breed and multiply.

### The Life-cycle of the Mosquito.

Starting from the adult fertilised female, the mosquito lays eggs which must be deposited on the surface of water. This accounts for the presence in large numbers of such insects round about, and inside, water-butts, water-tanks, and other such receptacles in gardens and backyards. The female mosquito descends lightly on to the surface of the water and deposits some hundreds of eggs glued together—in the case of the species under discussion—so as to form little boat-shaped or raft-shaped masses. Each egg has a lid on its under surface (the surface under the water), and in about two days this opens and lets out the larva, which then swims off and begins its life. Many species simply lay single eggs, that may be roughly united together to form patterns, or that just float about independently by themselves. The larva or wriggler (analagous to the caterpillar stage of the butterfly or moth) is divided into three parts—a head, thorax, and abdomen, the latter being long and containing at its posterior end the breathing apparatus through which the creature gets oxygen from the atmospheric air. In this species the shape of the breathing syphon is such that the creature when at the surface hangs down into the water almost at a right angle, but in other genera, anopheles for example, the breathing apparatus consists of two small holes on the back, this necessitating the creature lying flat or parallel with the surface of the water in order to get its air. Whichever position has to be adopted, it is essential for all mosquito larvæ to breathe air, and if we prevent this by pouring kerosene on the surface of the water, then the larvæ are suffocated and die. The larvæ feed on various algæ and other water plants and quickly grow if the weather is hot; in about 15 to 18 days they change into the pupa or chrysalis, a peculiar body shaped like a comma, which, unless one knew it or was told, one would certainly never associate with the previous larvæ. This is a resting stage, and no feeding is done now; breathing still goes on through the two breathing tubes which are now placed on the dorsal aspect of the head of the comma or thorax. In about two days the skin along this dorsal aspect splits down the middle and gradually the adult mosquito emerges. It rests on the cast-off pupal case for several minutes till its body and wings dry, and then rising lightly flies away.

### English Mosquitoes.

This is necessarily a very critical period of its existence, because the slightest puff of wind or movement of the water may wet it, and if this happens it cannot rise, but, gradually getting wetter, sinks down into the water and is drowned. Hundreds perish in this way, and in water that many pupæ have been in one always sees numerous dead adults that have been drowned. Those that escape—and, of course, for this calm, still weather is best—fly off, and probably enter some house, stable, outhouse, or other building. Here males that have also hatched out quickly find the females and fertilise them, and this seems to be about their only use, as they do not live very long, subsisting during their short spell of life on vegetable juices, and never sucking blood. In the tropics, at any rate, the next phase in the life-history is for the female to suck blood, some even going so far as to say that this meal is absolutely necessary, and that she cannot lay eggs without it. Certainly one notices that in mosquitoes kept in captivity abroad such a meal seems to stimulate the growth of the eggs in the ovaries and make the abdomen swell much more quickly; but after all it is probably only a purely acquired habit. It is extremely doubtful if it exists in England, as *Culex pipiens* only bites when the weather is really hot, which is seldom, and the *Anopheles maculipennis* is said not to bite at all, or seldom, in this country, though it is such a greedy feeder in Italy and the hotter countries of the South. Whichever happens then the abdomen of the mosquito gradually swells as the eggs develop, and, when properly matured, she seeks the surface of some water and deposits them there. In the tropics breeding goes on all the year round, the temperature being suitable at any time; but in England and other countries, where there is a winter, some other provision must be adopted for tiding over this period. This is accomplished by hibernation, which may take place either in the larval or adult stages.

Captive Insects.

As has been already stated the male mosquito does not suck blood; it lives on juices of plants and fruits. The females are very partial to blood, but life in their case can also quite readily be maintained by a purely vegetable diet. In the case of captive mosquitoes, bananas, apples, pears, dates, and solutions of sugar are the best foods to use, and water must also be supplied. The different genera and species vary greatly as to the ease with which they can be kept alive artificially. Anything in the slightest degree different from what they are accustomed to in Nature will quickly prove fatal; for example, one may construct a special house for this purpose, only to find later that none of the insects will live any time in it, even though in outhouses and cellars a few yards away they are numerous and healthy. A certain amount of heat is requisite, but if too great, and especially if too dry, then it does more harm than good. Feeds of blood are useful, but sometimes it is difficult to get them to partake of this, many evincing disinclination to bite at all.

Day Mosquitoes.

Mosquitoes are chiefly active at night, but such a rule is not absolute, certain genera coming out and biting in bright sunshine, and that even in the tropics. Most of them, however, love shade, and this applies to *Culex pipiens*, upon which this description is based. During the day, when the sun is shining, they rest in the shade of buildings, bushes, and trees. They fly about and are active all night, then they retire to roost again as the sun rises next morning. On dull days and in the shade of forests they move about during the day.

Fortunately mosquitoes have many enemies, but, even granting this, their numbers are often legion. They may be attacked at any stage of their life-history whether in the water or in the air. Fish are their chief enemies in the former, and destroy enormous quantities. If water tanks, fountains, butts, tubs, and other collections have a few fish put in them (minnows or gold fish, for example) then no mosquitoes will be allowed to develop there. The larvæ of some other insects (dragon flies, etc.), which also pass their first stages in water, also destroy them. As adults—swallows, bats, mature dragon flies, and other creatures prey upon them, while many harbouring in outhouses become entangled in spiders' webs and are eaten. Heavy thunder rains are very detrimental to any living in trees and bushes outside, and the combination of rainy weather with a sudden drop in the temperature is responsible for the death of millions.

